# Neurologic Presentation of Probable Seronegative Paraneoplastic Encephalitis in a Woman With an Ovarian Teratoma

**DOI:** 10.7759/cureus.8485

**Published:** 2020-06-07

**Authors:** Irina Chernyshkova, Bebsy Estefan, Md. Rezaul Hoque, Alice Lee

**Affiliations:** 1 Psychiatry, St. Barnabas Health System, Bronx, USA; 2 Psychiatry, St. Barnabas Medical Center, Bronx, USA; 3 Psychiatry, NewYork-Presbyterian/Weill Cornell Medical Center, Manhattan, USA; 4 Internal Medicine, St. Barnabas Hospital Health System / Albert Einstein College of Medicine, Bronx, USA; 5 Internal Medicine, City University of New York School of Medicine, Bronx, USA

**Keywords:** anti-n-methyl-d-aspartate, anti-nmda receptor encephalitis, limbic encephalitis, teratoma, seronegativity, myoclonus, seizures, memory, psychiatric, neurological symptoms

## Abstract

We describe a case of probable autoimmune encephalitis developed as a result of paraneoplastic syndrome in a woman with an ovarian teratoma. Patients may present with psychiatric and neurological symptoms, which are caused by anti-N-methyl-D-aspartate (anti-NMDA) receptor antibodies produced in response to a teratoma that crosses the blood-brain barrier and damages brain tissue in the limbic area, causing encephalitis. Our patient presented with seizures, myoclonus, and memory problems.

This is a relatively newly discovered and rare problem; however, it can be quite debilitating if left untreated. This diagnosis may be often missed due to the absence of highly sensitive tests. Autoimmune encephalitis has to be on the list of differential diagnoses for patients with new-onset psychiatric or neurological symptoms.

## Introduction

Anti-NMDA receptor (anti-NMDAR) encephalitis is paraneoplastic encephalitis that has been described to be associated with teratomas [1]. Paraneoplastic encephalitis is a type of autoimmune encephalitis affecting the limbic system [2]. Autoimmune encephalitis was first described in the 1960s as limbic encephalitis and was linked to herpes infection. The most common malignancy associated with paraneoplastic encephalitis is bronchial carcinoma, typically small cell carcinoma, but also rarely an intracranial neoplasm such as astrocytoma [3]. The first cases linking NMDA receptor encephalitis and ovarian teratoma were described in 2005 [4]. The average age of onset of symptoms is 21 years, although cases have been described in patients ranging from eight months to 85 years. Estimated mortality has been reported at about 7% [5]. More than 1000 cases have been reported in the past 10 years, with the most common association in a young woman being ovarian teratomas.

There have also been reports of recurrent teratomas with recurrent encephalitis while others have presented with encephalitis months to years following removal of the teratoma [6]. Regardless of the histologic type, teratomas that contain neural tissue could trigger an immune response resulting in the overproduction of anti-NMDAR antibodies [7]. Auto-antibodies are created against NMDA receptors on the surface of hippocampal neurons. Physiologically, NMDAR is important for higher functions such as learning and memory. Overactivation of the receptors may cause excitotoxicity, leading to the development of epilepsy, dementia, and stroke. On the contrary, low NMDAR activity results in psychiatric symptoms like schizophrenia [8]. The inhibition of NMDAR reduces gamma-aminobutyric acid (GABA) release, which inhibits glutamate release in the postsynaptic neurons. Glutamate is a major excitatory neurotransmitter in the brain, and diminished glutamate has been implicated in psychotic and neurocognitive symptoms in patients with schizophrenia [9]. The anti-NMDAR antibodies are the most common among neuronal cell-surface antibodies. They target the extracellular epitopes of synaptic receptors of components of synaptic proteins. Intracellular antibodies targeting nuclear or cytoplasmic proteins, such as anti-Hu, anti-ma, or anti-Ri, are associated with poorer prognosis [10]. Removal of the malignancy is associated with symptom relief; also, removal decreases the incidence of relapse of symptoms. Patients with positive anti-NMDAR antibodies generally responded well to treatment. Autoimmune encephalitis in the setting of teratoma is most common in young Asian and black females [4].

Since then, many case reports have been written and many of them present with psychiatric symptoms in combination with neurological symptoms. Our patient presented with predominantly neurological and cognitive features.

There have been cases describing symptoms that indicate autoimmune encephalitis, but the cerebrospinal fluid (CSF) and blood analysis is negative for antibodies. These cases have been called seronegative autoimmune encephalitis. A few theories were proposed to explain seronegativity in autoimmune encephalitis. One is that due to technical limitations and a subclinical picture of initial symptoms, the proper diagnosis was delayed, and by the time diagnosis was suspected, the number of antibodies had decreased below the threshold of detection. Especially in older individuals, the penetrability of the brain-blood barrier increases, which allows even undetectable levels of antibodies to cross and impede the brain function.

Another theory behind seronegativity is that there are antibodies that have not been discovered yet and are causing the dysfunction. Further research needs to be done to determine if other antibodies are causing similar symptoms, which may account for the seronegative cases. A third theory is that most frequently, the blood is checked for antibodies and not CSF. A recent study has shown that the anti-NMDAR antibodies were negative in the serum of 13% of cases with positive antibodies in the CSF [11-13]. Autoimmune encephalitis associated with neuronal surface antibodies is generally more likely to respond to immunotherapy, resulting in a good recovery in up to 70%-80% of cases [14].

The typical presentation is movement disorders after a flu-like prodromal phase. Other symptoms include rapidly progressive seizures, memory deficits, speech problems, and repetitive movement. Psychiatric symptoms like aggression, psychosis, delusions, and altered mood have also been described in the literature [15]. Patients often initially develop psychiatric symptoms followed by neurological manifestations. Typically, older patients present with acute behavioral changes, whereas younger patients present with movement disorder and seizures [4].

## Case presentation

Our patient is a 55-year-old female who was brought to the emergency room (ER) by emergency medical services (EMS) due to altered mental status. She was found face down, covered in urine and feces on her apartment floor. She was seen last time by her neighbors two days prior, in her usual state of health. What happened prior to the event is unclear because the patient appeared to have retrograde amnesia and confabulations.

Patient history

She had a history of a motor vehicle accident at the age of 39, which resulted in a concussion. Past medical history was significant for hepatitis C, asthma, and hypertension. The patient was also previously diagnosed with bipolar disorder and anxiety, with panic attacks. In addition, at the age of 47, she presented with her first episode of psychosis, which required inpatient psychiatric treatment (2011 and 2016) due to disorganized, bizarre behavior and paranoid ideations, unformed auditory hallucinations, and an increase in irritability and anxiety. She had a history of suicidal thoughts at ages 18 and 25 and was treated with Prozac and clonidine and currently denied intents, plans, or attempts of suicide. She used alcohol on weekends, one marijuana blunt every day, benzodiazepines (long-term clonazepam use), and methadone 25 mg daily. She was born and raised in New York. She completed high school and three semesters in college. She worked as a clerk in the past but is currently unemployed.

Lab results

Her leukocyte count was slightly increased to 13,400 on the first day but decreased to 6,600 within the first week.

At first, her laboratory studies were significant for a creatinine phosphokinase of 1541, sodium levels of 146, and lactic acid of 7.6. Her capillary blood glucose (CBG) was within normal limits. Her troponins were negative. Her carbohydrate antigen (CA) 19.9 was increased slightly at 41 (with the normal range being 0-35 U/mL) but cancer antigen (CA) 125 and carcinoembryonic antigen (CEA) were normal.

Her blood-alcohol and acetaminophen levels were undetectable, blood ammonia level was 52, lithium level <0.1, and valproic acid level <10.0. Her urine drug screen was positive for tetrahydrocannabinol (THC) and benzodiazepines but negative for barbiturates and cocaine. Her first blood cultures grew Staphylococcus (S.) epidermidis and S. hominis.

Computed tomography (CT) of her brain, cervical spine, thorax, and abdomen were negative for acute pathology. But CT of the pelvis showed a large complex mass in the left adnexa, presumably corresponding with a mature cystic ovarian teratoma.

A lumbar puncture for a routine CSF study and NeoEncephalitis panel was performed. The NeoEncephalitis panel included tests for the CASPR2 antibody, GAD65 antibody, LGI1 antibody, NMDAR (NR-1 subunit) autoantibody, VGK antibody, Recombx Amphiphysin autoantibody, Recombx Hu autoantibody, and Recombx MaTa autoantibody). CSF serology studies for encephalitis came back 14 days later, negative for any antibodies. CSF was also checked for bacterial culture sensitivity, acid-fast bacilli (AFB) culture, fungal culture, and sensitivity, West Nile virus antibody, cryptococcal Ag, herpes simplex virus (HSV)-1 and HSV-2 deoxyribonucleic acid (DNA), and enterovirus polymerase chain reaction (PCR). In her CSF, three white blood cells (WBCs) were found in tube # 1 and six WBCs in tube # 4, which indicated the absence of pleocytosis. All other tests came back negative.

Table 1 shows the results of the CSF study.

**Table 1:** CSF study results CSF: cerebrospinal fluid; WBC: white blood cell; RBC: red blood cell; HSV: herpes simplex virus; RT-PCR: reverse transcription-polymerase chain reaction; AFB: acid-fast bacilli

Appearance	Clear
Xanthochromia	Negative
WBC	6 (Tube # 4), 3 (Tube # 1)
RBC	1093 (Tube # 4), 13 (Tube # 1)
Neutrophil	10
Lymphocyte	86
Monocyte	3
Eosinophil	1
Glucose	67
Protein	21
NeoEncephalitis panel	negative for all
HSV-1 DNA	Negative
HSV-2 DNA	Negative
Enterovirus RT-PCR	Negative
Fungus culture	Negative
Cryptococcal Ag	Negative
AFB culture	Negative
West Nile Ab	Negative

Her magnetic resonance imaging (MRI) brain with contrast revealed no acute intracranial pathology. There was no acute large territorial infarct, midline shift, mass effect, or acute hydrocephalus. A few scattered foci of T2 prolongation were seen involving the subcortical centrum semiovale and corona radiata, without abnormal enhancement. There was no abnormal parenchymal or leptomeningeal enhancement. No acute or chronic blood breakdown products were seen. The major vessel flow voids were identified. The sella and pineal regions appeared unremarkable. No evidence of cerebellar tonsillar herniation was noted.

Hospital course

In the ER, she presented disheveled, alert, oriented x2/3; at that time, she was able to move all extremities, however, she had jerky movements of all four extremities. Upon arrival at the ER, she had one episode of a tonic-clonic seizure that responded well to lorazepam. However, she continued to have involuntary, continuous shaky movements in bilateral upper and lower extremities and tongue fasciculations. Her tremor was aggravated by attempting to perform any activities. Tremors did not interfere with her speech. She did not have any sensory deficits. Her vitals were all within normal limits. Also, upon arrival to the ER, she had stage 1 pressure ulcers on her extremities. Her right eye had a conjunctival injection and left eye with periorbital ecchymosis and frank purulence. These could be signs that she was unconscious for a long time or suffered an assault (which was one of the differential diagnoses at that time).

She was admitted to the care of the intensive medical care unit (IMCU) due to altered mental status and bacteremia. While on the unit, she was unable to perform basic activities of daily living (ADLs) such as using utensils to feed herself, walk, lift herself to sit, or turn in her bed secondary to the tremors. She was started on vancomycin for bacteremia, which was discontinued when her repeat blood culture did not grow those bacteria. It deemed her first blood culture was contaminated with normal skin flora. When her mental status improved, she was transferred to the floor.

On her ninth day of hospitalization, she was started on intravenous (IV) dexamethasone 4 mg three times a day for suspected autoimmune encephalitis. Her tremors decreased dramatically after starting the steroids. She was able to hold and use her phone. On the third day of steroid treatment, she was able to feed herself using utensils. Later, she was able to sit on a chair and walk with the help of a physical therapist. A laparoscopic bilateral salpingo-oophorectomy and a cystoscopy were performed without complications four days after she started dexamethasone. The patient tolerated the procedure well and did not have any post-surgical complications. She was given dexamethasone in a tapering dose.

On Day 3 of therapy with steroids, her dexamethasone dose was decreased to 2 mg three times daily from 4 mg two times daily. The patient’s condition worsened slightly the following day. While she was able to sit up before, her intention tremors worsened, and she was no longer able to sit up. Tremors reduced when her previous dose was resumed. She was discharged to a short-term rehabilitation center to continue recuperation.

The patient has been following up to our neurology clinic since discharge from the hospital. On her first visit, she was still noted to have ataxia, although it was improving. So, she was put back on her previous dose. On her second follow-up visit, she was noted to have slight scanning speech and ocular dysmetria. So, her steroid dose was lowered to 1 mg daily. She will continue to follow up in the neurology clinic.

The atypical older age of onset and the initial seronegativity of her CSF and serum encephalopathy panels led to a slow workup of this case. Over time, several factors led us to conclude the diagnosis of possible seronegative NMDAR encephalitis, including the presence of ovarian teratoma, therapeutic response to immunotherapy, and significant improvement of symptoms after tumor removal.

Differential diagnoses in our case included trauma due to domestic violence (staff in her housing were concerned about a possible assault by her significant other), intracranial hemorrhage, ischemic episodes, benzodiazepine withdrawal, potential overdose, alcohol withdrawal seizures, and post-concussion syndrome. Rapid improvement of her symptoms on steroids ruled out post-anoxic myoclonus as the most common cause of intention myoclonus. The trauma of domestic violence was suspected due to extensive bruising and lesions on our patient’s extremities and face. However, on further examination, her injuries were consistent with trauma from a fall and seizures. The chronology of events told by the patient changed multiple times during the course of her stay. The discrepancies in her story could be a symptom of the suspected autoimmune encephalitis, which could manifest as confabulation or retrograde memory loss. Intracranial hemorrhage and ischemic episodes were suspected because of how suddenly her symptoms manifested; however, this was ruled out by the CT brain, which did not show acute pathology. The patient was on long-term benzodiazepines and withdrawal could manifest with seizures; she was given clonazepam in the hospital, which did not alleviate her symptoms. Urine toxicology screen was performed and was positive for THC and benzodiazepines six days after her arrival at the hospital. Benzodiazepines in her urine were most likely from the doses in the ED for the seizure. The toxicologist opinioned that clonazepam withdrawal was the most likely etiology of the seizure given the absence of lithium and valproate in blood and limited history obtained from the patient. The finding of the teratoma pointed toward autoimmune encephalitis, even in the setting of seronegativity.

## Discussion

The majority of teratomas are asymptomatic. Immune-mediated paraneoplastic syndrome associated with teratomas may be the only presenting symptom. However, the symptoms, as well as progression, vary greatly among cases. There were cases that reported patients who had psychiatric syndromes or a combination of both psychiatric and neurologic symptoms. Some reports show that the symptoms may have been preceded by subsyndromal levels, so the length of the symptoms is unclear. Furthermore, seronegativity may further delay the diagnosis. One case report noted a one-year history of subclinical psychiatric symptoms before the symptoms that prompted an emergency room visit. Very few seronegative cases have been reported in the literature, with the majority of cases starting at younger ages. Most studies are retrospective cohorts and there are no randomized controlled trials. Given the retrospective uncontrolled data, the literature has an inherent bias, including severity and reporting bias. It must be kept in mind that patients with ovarian teratoma could develop other forms of encephalitis without NMDAR antibodies and that any adolescent or young adult, particularly female, who develops subacute brainstem-cerebellar symptoms or opsoclonus-myoclonus that is suspected to be immune-mediated (because of the rapid onset of symptoms and/or CSF pleocytosis) should be investigated for teratoma of the ovary (or testes in male patients) [16].

Usually, serology tests take up to two weeks to yield results and in some facilities, tests are not rightly available. This condition requires a fast diagnosis and treatment to ensure the best outcomes. Table 2 describes the proposed diagnostic criteria in the absence of serology results [15].

**Table 2:** Diagnostic criteria Reference [15] EEG: electroencephalography, CSF: cerebrospinal fluid, MRI: magnetic resonance imaging

Requires all three criteria to be present
Subacute onset of cognitive deficits, neurological, or psychiatric symptoms
Bilateral brain abnormalities in medial temporal lobes in T2 weighted MRI images
CSF pleocytosis or EEG with epileptic discharges or slow-wave activity involving the temporal lobes
Symptoms not better explained by other conditions

The diagnosis of limbic encephalitis is based on clinical presentation and lab results such as MRI, electroencephalography (EEG), and lumbar puncture. This is because antibody tests can take time to get results back and there are cases where the patient is seronegative. Antibody detection in the CSF is not required to consider the diagnosis of autoimmune limbic encephalitis. Data shows that 7%-26% of autoimmune limbic encephalitis cases are seronegative [17]. Seventy-four percent of cases in seronegative limbic encephalitis had an abnormal MRI [17]. Other studies, including EEG, fluorodeoxyglucose positron emission tomography (FDG PET), and single-photon emission computerized tomography (SPECT) testing were not done in this case but could have aided in diagnosis.

What complicated this case was that our patient was seronegative on a NeoEncephalitis panel and her MRI did not show acute changes. The differential diagnosis that was not considered by the team at that time but Hashimoto encephalitis was possible. It presents with the following symptoms: (1) Encephalopathy with seizures, myoclonus, hallucinations, or stroke-like episodes; (2) Subclinical or mild overt thyroid disease (usually hypothyroidism); (3) Brain MRI normal or with non-specific abnormalities; (4) Presence of serum thyroid (thyroid peroxidase, thyroglobulin) antibodies; (5) Absence of well-characterized neuronal antibodies in serum and CSF; 6. Reasonable exclusion of alternative causes.

The consensus is to start treatment with an immunosuppressive as soon as possible. This is because the disease progresses quickly and delaying immunotherapy could have poor long-term outcomes. First-line therapy is corticosteroids, intravenous immunoglobulin, and plasma exchange. Second-line therapy can be initiated if first-line therapy does not have an appreciable response and include rituximab and cyclophosphamide. Also, finding a tumor and its removal has shown to improve symptoms in over 50% of patients within the first four weeks and almost all patients within two years. The literature also suggests that some patients relapse within two years despite immunotherapy even if initial improvement is seen after tumor removal [1]. Although the majority of encephalitis with neuronal surface antibodies are treatment-responsive, anti-IgLON5 encephalitis appears to be different from the other autoimmune encephalitis, with poor response to immunotherapy and high mortality [7]. In contrast, treatment resulting in full recovery is not uncommon, particularly with ovarian teratomas [18]. Our patient's improvement on just one day of steroids was drastic.

**Figure 1:**
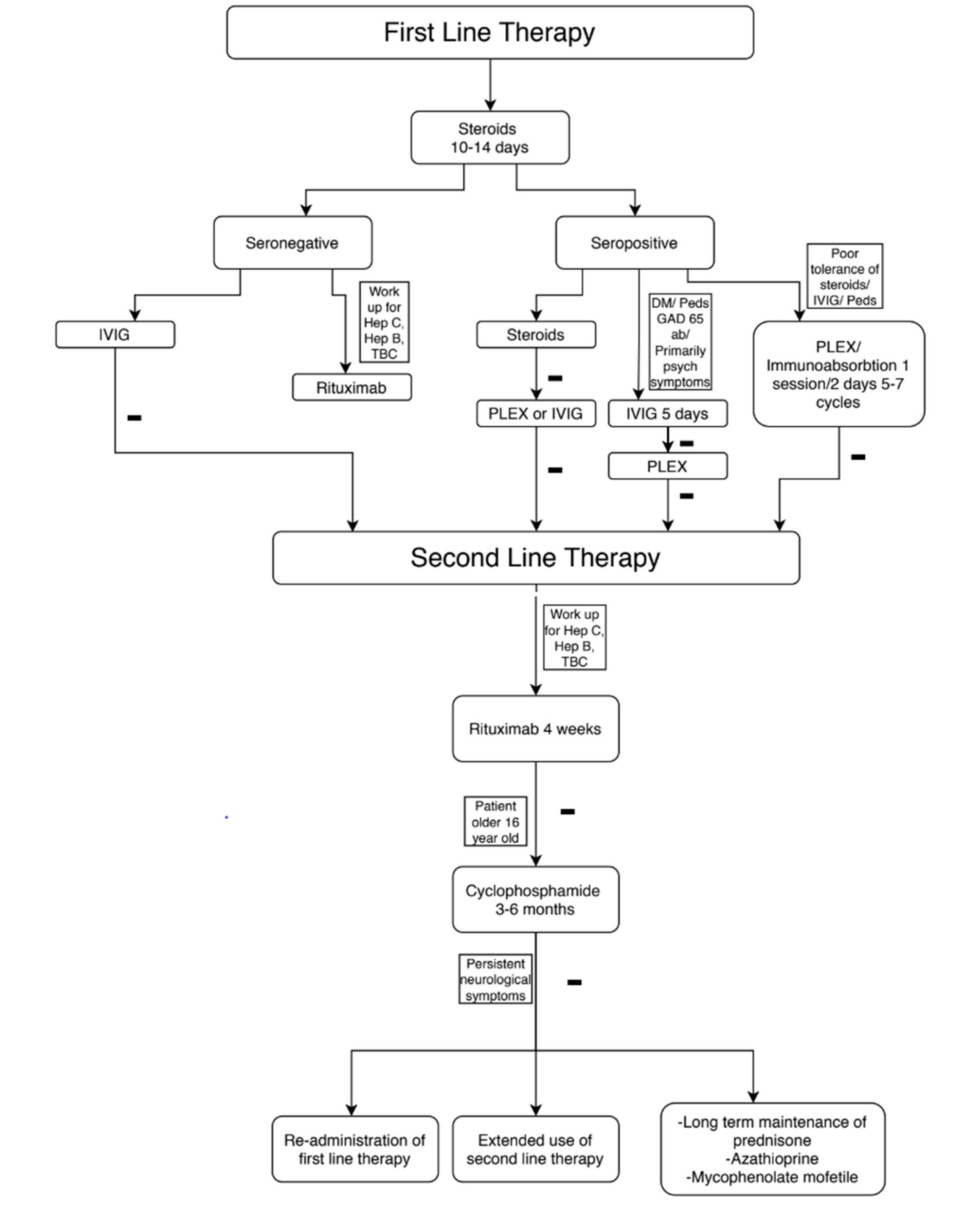
Stepwise approach in treatment: Summary TBC: tuberculosis, Hep C: hepatitis C, Hep B: hepatitis B, DM: diabetes mellitus, Peds: children, Psych: psychiatric, GAD65 ab: glutamic acid decarboxylase antibody, IVIG: intravenous immunoglobulin, PLEX: plasma exchange therapy

The demographic where suspicion of paraneoplastic encephalitis should be raised are females of African or Asian descent. The median age of presentation is 21. However, the discovery of paraneoplastic encephalitis is relatively new and there are cases in the literature that describe patients who have the disease and do not fit the demographic. If paraneoplastic encephalitis is suspected on MRI, EEG, lumbar puncture (LP), and the NeoEncephalitis panel should be done. The suggested treatment has to be started as early as possible. If the first-line treatment fails after 10-14 days, which could be presented as a relapse of symptoms, absence of response, or severe disease, second-line therapy should be initiated. There are special recommendations for certain treatments that we came across during the literature review. Immunoadsorption for the treatment of AE demonstrates a 64% decrease in CSF antibody titers at early follow-up. Rituximab showed to be more effective in seronegative cases. Cyclophosphamide treatment is recommended for patients older than 16 years of age. Medications and a stepwise approach to their administration are presented in Table 3 and Figure 1.

**Table 3:** Medications, doses, and length of treatment Reference: [4] IV: intravenous, IVIG: intravenous injection of immunoglobulin, CSF: cerebrospinal fluid, IL-2: interleukin 2

First-Line Therapy up to 10-14 Days	Corticosteroids (Methylprednisolone)	1 g Daily, for 3-5 Days
	Immunoglobulin	IV 2 g/kg over 5 days (400 mg/kg/day)
	Plasmapheresis	1 session every other day for 5-7 cycles
	Immunoadsorption	
Second-line therapy (immunomodulatory agents)	Rituximab	375 mg/m x 2 weekly IV infusion for 4 weeks
	Cyclophosphamide	750 mg/m x 2 monthly for 3-6 months
Alternative therapy	Re-administration of first-line therapy	
	Extended use of second-line therapy	
	Long-term maintenance with prednisolone, azathioprine or mycophenolate mofetil	
	Tocilizumab	Initially, 4 mg/kg, followed by an increase to 8mg/kg monthly based on clinical response
	Low-dose IL-2 therapy and Treg modulation	1.5 million IU/ day, 4 subcutaneous injections with a 3-week interval
	Bortezomib	
Steroid-sparing agents used for maintenance therapy	Azathioprine	Initially, 1-1.5 mg/kg once daily or divided twice daily, target 2-3 mg/kg/d
	Mycophenolate mofetil	Initially 500 mg twice daily, target 1000 mg twice daily

The prognosis of anti-NMDAR encephalitis is usually better than other types of paraneoplastic encephalitis. It is reported that approximately 75% of patients recovered completely or only suffered minor disabilities. Compared to other types of paraneoplastic encephalitis, anti-NMDAR encephalitis differs because it results in a highly specific syndrome, is associated with benign tumors, usually affects young women, and has a good prognosis with early treatment [19].

Imaging should be done and a meticulous search for the tumor and removal would be the definitive treatment. Patients may continue to have symptoms after the removal of the tumor so continued maintenance therapy is recommended to decrease the risk of relapse.

## Conclusions

When patients present with the first episode of psychosis, especially when unresponsive to antipsychotics or with neurologic symptoms, it is important to consider the diagnosis of paraneoplastic encephalitis. The clinical presentation is variable so diagnosis can be difficult and subsequent treatment may be delayed. In addition to the variable presentation, patients frequently have other comorbidities that can further slow diagnosis. The consensus in the literature is to start immunotherapy treatment as early as possible. Our patient improved drastically immediately after starting treatment. If the patient has a tumor, definitive treatment is the removal of the tumor. Symptoms length is also variable but may be related to how quickly immunomodulating therapy was started. Cases have been described where patients relapse and are again symptomatic after improving with treatment. Further studies are required to investigate the best treatment approaches.
